# The Mediating Role of Cognitive Function in the Relationship Between Physical Performance and Depressive Symptoms Among Older Adults in China: A Prospective Longitudinal Study

**DOI:** 10.1155/nrp/1203145

**Published:** 2025-12-30

**Authors:** Na Zhang, Fang Wang, Manlan He, Lu Chen

**Affiliations:** ^1^ Department of Nursing Research Institute, Nanjing Drum Tower Hospital, Affiliated Hospital of Medical School, Nanjing University, Nanjing, Jiangsu, China, nju.edu.cn; ^2^ Department of Neurosurgery, Nanjing Drum Tower Hospital, Affiliated Hospital of Medical School, Nanjing University, Nanjing, Jiangsu, China, nju.edu.cn

**Keywords:** cognitive function, depressive symptoms, elderly, mediation effect, physical performance

## Abstract

**Background:**

Depressive symptoms in the elderly have become a growing public health problem as the population ages rapidly and the imbalance between supply and demand of mental health resources exists. This study aims to explore the predictive power of physical performance on depressive symptoms in Chinese older adults, examine the mediating role of cognitive function, and analyze its variations across genders.

**Methods:**

In this study, we included 3779 older adults aged 60 years and above who participated in the 2015 China Health and Retirement Longitudinal Study and completed follow‐up in 2018. We employed binary logistic regression and three‐knotted restricted cubic spline regression to examine the association between physical performance and depressive symptoms, assessed the mediating effect of cognitive function using the SPSS PROCESS macro, and analyzed the data separately in male and female subgroups.

**Results:**

The development of depressive symptoms during follow‐up (*n* = 982) was preceded by significantly lower physical performance scores at baseline. After adjusting for confounding factors, the presence of the lowest baseline short physical performance battery (SPPB) scores was independently associated with an increased risk of incident depressive symptoms (OR, 1.972; 95% CI: 0.91–4.26), similar to the trend of restricted plots. Meanwhile, cognitive function is an intermediate variable between physical performance and depressive symptoms, and the mediating effect of men is stronger, at 14.92%.

**Conclusion:**

This study confirms that poor physical performance is independently associated with depressive symptoms in Chinese older adults at the 3‐year follow‐up and further reveals that cognitive function serves as a mediator in this association with gender‐specific differences. These findings provide an important theoretical basis and potential clinical value for implementing targeted cognitive interventions in populations with declining physical function to alleviate depressive symptoms.

## 1. Introduction

Depression is a chronic condition characterized by persistent sadness and loss of interest or pleasure in previously rewarding or enjoyable activities [1]. As one of the leading causes of disability worldwide, it significantly contributes to the global burden of disease [2]. Concurrently, the accelerating pace of population aging presents multifaceted challenges in economic, social, and health domains [3]. Against this backdrop, late‐life depression has become increasingly prominent—according to the World Health Organization (2017), approximately 5 million older adults worldwide suffer from late‐onset depression. With the progression of population aging and the growing imbalance between supply and demand for mental health services, depression among older adults has evolved into a pressing public health issue requiring urgent attention.

Depressive symptoms serve as early warning signs and are associated with an increased risk of future depression. Timely intervention at this stage may help prevent the onset of subsequent mental disorders. This is especially crucial for older adults, whose physiological functions and mental health are closely interrelated. In the older adult population, physiological function and mental health of the elderly are closely interrelated [4]. Research indicates that declines in physical performance not only predict unexpected disability but also increase the risks of hospital readmission and mortality [5, 6]. Although previous studies have explored the association between depressive symptoms and physical function, confirming that depressive symptoms can lead to deterioration in physical performance [7–9], most such investigations have been conducted in non‐Hispanic populations. Consequently, the predictive value of physical performance for depressive symptoms in Chinese older adults warrants further validation.

In addition, approximately 8%–30% of older adults present with both cognitive impairment and depression [10, 11]. Memory, executive function, and processing speed examinations are helpful to find cognitive decline in older adults who have depressive symptoms [12]. Additionally, accumulating evidence suggests an association between physical performance and cognitive ability. For instance, Nicola Veronese found that poor short physical performance battery (SPPB) performance and slower gait speed predicted the new appearance of cognitive impairment at the follow‐up [13]. However, another study indicated there were no associations between skeletal muscle mass with cognitive performance in Type 2 diabetes [14]. Although some studies have reported on the association between sarcopenia, depressive symptoms, and cognitive function [15–18], their results investigated the relationships of sarcopenia with depressive symptoms and cognitive impairment in cross‐sectional study rather than longitudinal study. A recent longitudinal study on sarcopenia and depression among middle‐aged and older Chinese adults identified low physical performance as a risk factor for depression [19]. A study demonstrated that better cognitive performance in older adults was associated with better physical performance and lower depressive symptoms [20]. Moreover, physical activity enhances cognitive function indirectly by mediating depressive symptoms [21]. Additionally, a stronger correlation between severe depressive symptoms and cognitive impairment was observed in older Chinese males [22]. However, the gender differences in this association have not been fully explored. More importantly, the interactive mechanisms underlying these conditions remain unclear, particularly regarding the potential mediating pathway of “physical performance, cognitive function, and depressive symptoms,” which has not been thoroughly investigated.

In the light of this, the present study employs a longitudinal design to systematically examine the effect of physical performance on the risk of depressive symptoms after 3 years among Chinese older adults, with a specific focus on assessing the mediating role of cognitive function in this association. The findings are expected to elucidate the underlying mechanisms and offer new insights for early identification and prevention of depression in community‐dwelling older adults in China.

## 2. Methods

### 2.1. Participants

We extracted data from the China Health and Retirement Longitudinal Study(CHARLS),​ a nationally representative micro and longitudinal survey of Chinese people aged 45 and older years, which has been described elsewhere [23]. The survey recruited 17,708 participants in 10,257 households from 28 provinces in China at baseline in 2011–2012 and then followed up on the participants in 2013,2015, and 2018. Our study selected participants in 2015 as baseline and followed up in 2018. All participants were interviewed face‐to‐face to collect information about sociodemographic, health‐related information, and lifestyle factors using a standardized questionnaire. The survey study was approved by the institutional review board of Peking University (IRB00001052‐11015).

**Figure 1:**
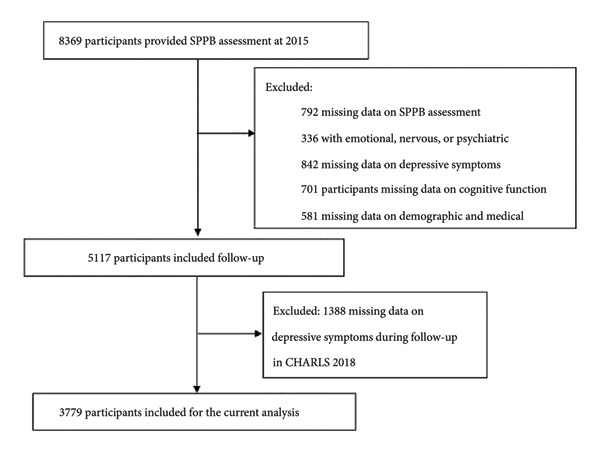
Flow of participants’ selection.

According to the purpose of the study, we selected the elderly aged over 60 years; no emotional, nervous, or psychiatric problems; demographic data such as gender, age, village or city, level of education, marital status, hypertension, dyslipidemia, diabetes, heart disease, stroke, number of diseases, accidental injury, activities, smoking status, drinking status, and body mass index (BMI); the depressive symptoms scores, the SPPB scores, and the cognitive function scores. Missing values and those that did not meet the inclusion criteria will be excluded. Based on these inclusion criteria, 3779 participants were selected from the CHARLS database. Screening of participants in this study is shown in Figure 1.

### 2.2. Depressive Symptoms

Each survey of the CHARLS used the Center for Epidemiological Studies Depression (CES‐D) scale to measure depressive symptoms. The CES‐D scale is used to assess the frequency of these feelings in the past week, and it includes 10 items: felt depressed, fearful, hopeful, unhappy, lonely, could not get going, bothered by little things, trouble concentrating, everything was an effort, and sleep was restless, with each item scoring from 0 to 3 points for a total of 30 points, higher score means a higher depression level. This scale has good reliability and validity. Depressive symptoms are considered if total scores ≥ 12 on the CES‐D scale, and this cutoff value was validated in a Chinese older adult population [24].

### 2.3. Physical Performance

Physical performance was measured via SPPB. This is a widely used scale to explore declines in physical performance in older adults [25]. It includes the following: balance tests (semitandem, full‐tandem, and side‐by‐side): full‐tandem 10 s or longer (4 points), full‐tandem 3–9 s (3 points), semitandem 10 s and full‐tandem 0–2 s (2 points), side‐by‐side 10 s and semitandem < 10 s (1 point), and unable to perform the tests (0 points); walking speed: score at a faster pace, ≥ 0.78 m/s (4 points), 0.61–0.77 m/s (3 points), 0.44–0.60 m/s (2 points), and ≤ 0.43 m/s (1 point), and unable to perform the test (0 points); and five repetitions of standing up from the chair: ≤ 11.1 s (4 points), 11.2–13.6 s (3 points), 13.7–16.6 s (2 points), ≥ 16.7 s (1 point), and unable to perform the test (0 points) [26]. The scores of these three components are added together to give an overall score ranging from 0 to 12. To avoid the measurements error, before the test, each visitor is trained and qualified. We classified participants into high dysfunction (SPPB score < 4/12), moderate dysfunction (4/12 to 7/12), and low dysfunction (score ≥ 8/12) based on the cutoff point of clinical significance for older adults [27].

### 2.4. Cognitive Function

The CHARLS team verified the effectiveness of the cognitive test scale in the Chinese elderly, and many studies have described this [28]. Three composite measures of cognitive function were used in this study: orientation and attention, episodic memory, and visual construction. The first of these, orientation and attention, comprises judging whether participants correctly answered the year, month, day, season, week, and subtracted 7 from 100 for 5 consecutive times, for a total of 10 points; episodic memory was measured by participants reading 10 random words immediately (within 2 min) and then delayed (4–10 min later) to repeating as many words as possible. In line with previous studies, the score of episodic memory was calculated by the average of immediate recall and delayed recall scores. Visual construction was assessed through a task in which participants redrew the displayed map. The total score of cognitive function was 21 points, and higher scores indicated better cognitive function. In this study, we used the cognitive function assessed at baseline.

### 2.5. Statistical Methods

Data cleaning and statistical analysis were performed using Stata 15.1, SPSS 26.0, and R 3.6.1. Baseline characteristics were summarized as means ± standard deviations (SDs) or proportions, as appropriate. Differences in characteristics between participants with and without depressive symptoms were compared using *t*‐tests, chi‐square tests, or other appropriate statistical methods.

The association between depressive symptoms and physical performance was examined using binary logistic regression, with results expressed as adjusted odds ratios (ORs, an estimate of the strength and direction of the relationship) and 95% confidence intervals (CIs, to measure the precision and statistical reliability of the estimate). To further verify the robustness of the results, multiple linear regression was employed. Additionally, a restricted cubic spline regression model was used to assess the nonlinear relationship between baseline physical performance scores and depression risk after 3 years.

**Figure 2:**
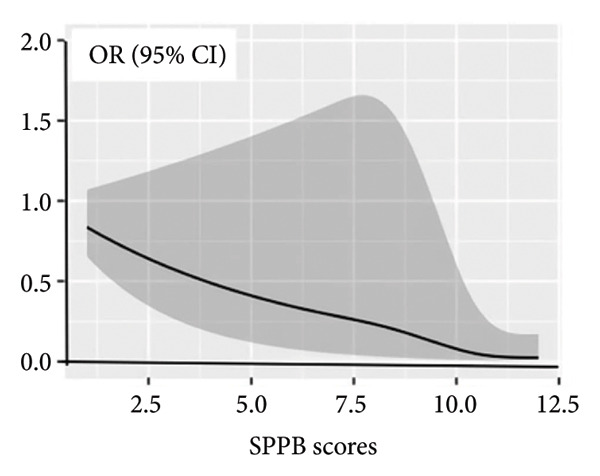
Associations between physical performance and future depressive symptoms. Lines are based on a multivariable restricted cubic spline regression of OLS regression, adjusted for age, gender, BMI, educational level, residence, marital status, accidental injury, number of diseases, activities, smoking status, and drinking status. The SPPB score ranges from 0 to 12, with the highest score representing the lowest risk of disability.

Pearson correlation analysis was applied to evaluate associations between continuous variables. Mediation analysis was performed using the SPSS PROCESS macro to examine whether cognitive function mediated the relationship between physical performance and depressive symptoms. Separate analyses were conducted for male and female samples to explore gender differences. Figure 2 was described by GraphPad Prism 9.1.1 (GraphPad Software Inc., San Diego, CA, USA). The significance level was set as 0.05 in this study.

## 3. Results

There are 3779 participants (53.11% men) in the final analysis, with an average age of 66.84 ± 5.47 years. Among all participants, after 3 years, 982 (25.98%) participants reported with depressive symptoms. In comparison with participants without depressive symptoms, the participants with depressive symptoms were more likely to be female, lower levels of education, live in rural areas, rarely join in activities, poorer cognitive function, and physical performance as shown in Table 1.

**Table 1:** Participant characteristics at baseline with or without depressive symptoms.

			**Depressive symptoms**		**p** **value**
		**Total**	**With (*n* = 982)**	**Without (*n* = 2797)**	

Gender	Male	2007 (53.11)	392 (39.92)	1615 (57.74)	≤ 0.001
	Female	1772 (46.89)	590 (60.08)	1182 (42.26)	

Village or city	City	678 (17.94)	103 (10.49)	575 (20.56)	≤ 0.001
	Rural	3101 (82.06)	879 (89.51)	2222 (79.44)	

Education level	No formal education	944 (24.98)	331 (33.71)	613 (21.92)	≤ 0.001
	Primary school	1872 (49.54)	505 (51.43)	1367 (48.87)	
	Middle or high school	897 (23.74)	140 (14.26)	757 (27.06)	
	College or above	66 (1.75)	6 (0.61)	60 (2.15)	

Marital status	Married	3170 (83.88)	774 (78.82)	2396 (85.66)	≤ 0.001

Number of diseases	0	1168 (30.91)	299 (30.45)	869 (31.07)	0.636
	1	992 (26.53)	261 (26.58)	731 (26.14)	
	2	721 (19.08)	177 (18.02)	544 (19.45)	
	≧ 3	898 (23.76)	245 (24.95)	653 (23.35)	

Accidental injury	Yes	294 (7.79)	82 (8.35)	212 (7.59)	0.441

Activities	No	1672 (44.24)	436 (44.40)	1236 (44.19)	0.011

Smoking status	Still	1075 (28.45)	285 (29.02)	790 (28.24)	0.879
	Quit	505 (13.36)	132 (13.44)	373 (13.34)	
	Never	2199 (58.19)	565 (57.54)	1634 (58.42)	

Drinking status	Still	1358 (35.94)	356 (36.25)	1002 (35.82)	0.683
	Quit	425 (11.25)	103 (10.49)	322 (11.51)	
	Never	1996 (52.82)	523 (53.26)	1473 (52.66)	

Age		66.84 ± 5.47	66.90 ± 5.41	66.83 ± 5.49	0.732

BMI		23.55 ± 3.54	23.18 ± 3.57	23.69 ± 3.52	≤ 0.001

Cognitive function		10.37 ± 3.99	8.96 ± 3.94	10.86 ± 3.90	≤ 0.001

Physical performance		10.53 ± 1.84	10.07 ± 2.03	10.70 ± 1.74	≤ 0.001

*Note:* Values are mean ± SD, *n* (%).

Abbreviation: BMI = body mass index.

Over a maximum follow‐up of 3 years, 982 (25.98%) participants had depressive symptoms. We used multiple linear regression to explore the relationship between physical performance and depression and found that it was statistically significant (*p* < 0.001). The results are shown in Table 2. Analyzing depressive symptoms as a binary variable and physical performance as a continuous variable revealed a statistically significant nonlinear relationship (*p* for nonlinearity = 0.004), suggesting a curvilinear rather than a simple linear association, as shown in Figure 2.

**Table 2:** Multiple linear regression analysis of factors influencing depressive symptoms.

Variable	*B*	*t*	*p*	95% CI
Intercept	25.261	13.805	< 0.001	21.673, 28.848
Gender	1.103	0.212	< 0.001	0.686, 1.520
Age	−0.101	0.019	< 0.001	−0.138, −0.064
Education	−0.135	0.158	0.394	−0.445, 0.175
Marital status	1.029	0.275	< 0.001	0.490, 1.569
Number of diseases	−0.029	0.085	0.731	−0.196, 0.137
Activities	−0.032	0.096	0.741	−0.220, 0.156
Smoking status	−0.278	0.121	0.022	−0.515, −0.040
Drinking status	−0.119	0.116	0.303	−0.347, 0.108
BMI	−0.157	0.028	< 0.001	−0.213, −0.102
Cognitive function	−0.342	0.029	< 0.001	−0.399, −0.285
Physical performance	−0.459	0.057	< 0.001	−0.570, −0.347

**Figure 3:**
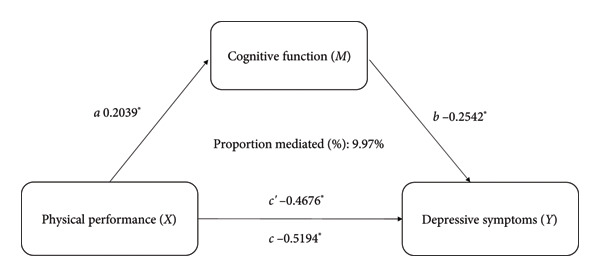
Cognitive function as a mediator of the physical performance and depressive symptoms, adjusted for age, gender, BMI, educational level, residence, marital status, accidental injury, number of diseases, activities, smoking status, and drinking status. The indirect effect of *X* on *Y* was significant (*ab* = −0.0518, SE = 0.0106; 95% CI, −0.0734∼−0.0323). *c* = *c*′ + *ab*. ^∗^ *p* < 0.001.

The Pearson correlation analysis​ showed negative significant relationships between depressive symptoms of cognitive function (*r* = −0.253, *p* < 0.01) and physical performance (*r* = −0.168, *p* < 0.01). Moreover, there was a positive significant association between cognitive function and physical performance (*r* = 0.206, *p* < 0.01). Mediation analysis was used to explore whether cognitive function is an intermediate variable between physical performance and depressive symptoms, and the results showed that cognitive function explained 9.97% (indirect effect *β* = −0.468, 95% CI [−0.582, −0.353]) of the association between physical performance and depressive symptoms (shown in Figure 3). In this model, the size of the indirect effect of the mediation through cognitive function was dependent on the moderator gender with smaller effects in women (3.57%, indirect effect *β* = −0.317, 95% CI [−0.462, −0.251]) than in men (14.92%, indirect effect *β* = −0.587, 95% CI [−0.667, −0.371]), as shown in Figure 4.

Figure 4Cognitive function as a mediator between physical performance and depressive symptoms in different genders, adjusted for age, BMI, educational level, residence, marital status, accidental injury, number of diseases, activities, smoking status, and drinking status. *C*′ = direct effect, *c* = *c*′ + *ab*. ^∗^ *p* < 0.05. (a) Male and (b) female.

**Figure:**
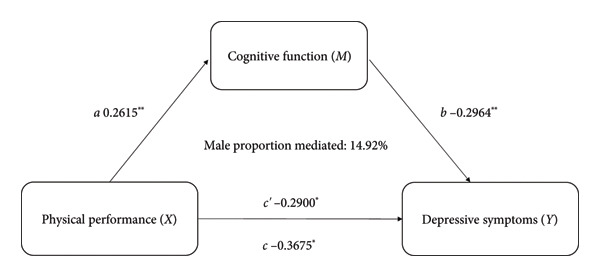
(a)

**Figure:**
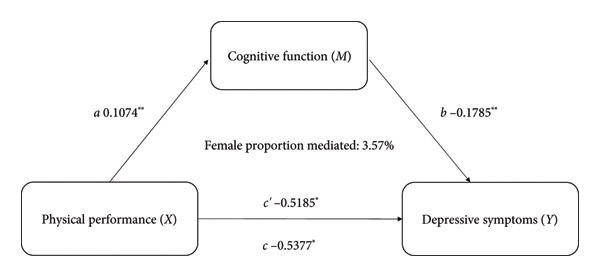
(b)

## 4. Discussion

We found that baseline cognitive function and physical performance were correlated with follow‐up 3‐year depressive symptoms. After controlling for confounding factors, baseline poorer physical performance was associated with a higher incidence of depressive symptoms. Also, cognitive function partially mediated the association between physical performance and depressive symptoms. In this model, the size of the indirect effect of the mediation through cognitive function was dependent on the moderator gender: smaller effects in women than in men.

China’s rapid urbanization and the trend toward nuclear families are weakening the traditional multigenerational support system, exacerbating the psychological adaptation challenges faced by older adults with declining physical function. These findings are consistent with previous research, with participants scoring the lowest in SPPB having an increased odd (2.7‐fold) of developing depression [29]. Similar to their study, we found that the presence of lowest baseline SPPB scores was independently associated with an increased risk of incident depressive symptoms (OR, 1.972). Several reasons may account for this phenomenon: first, low physical performance may reduce functional mobility and increase the risk of falls [30]; fear of falling was negatively correlated with the volume of brain regions important for emotional control, motor control, executive function, and visual processing [31]. Second, low physical activity may increase the risk of depression through the dual pathways of elevated oxidative stress and reduced sex hormones [32, 33]. Third, people with poor physical performance may be more socially isolated, and there is growing evidence linking social isolation to depression [34, 35]. Prospective cohort studies need to further elucidate the relationship between low physical activity, fear of falling, and social isolation with brain structure and depression.

In our study, the mediation analysis found that cognitive function plays a role in the association between physical performance and depressive symptoms, which may be because people with poor cognitive function are prone to negative emotions [36, 37]. Previous studies have shown that the presence of depressive symptoms is associated with cognitive function [38, 39], but the exact nature of the link between cognitive decline and depression is not entirely clear. A recent study indicated that compared to nutritional status, depression is even more related to cognitive function and physical performance [40]. This may be because older adults with deteriorating cognitive function may also have thoughts of deteriorating functional status, and cognitive impairment may be aggravated, thus worsening the ability to perform daily activities [41]. Unlike their cross‐sectional study, we used a prospective cohort study to confirm that underlying physical performance predicts future depression and that cognition plays a moderating role.

In addition, we found that cognitive function is a partial mediator of the relation between physical performance and depressive symptoms in this model. The size of the direct effect of the mediation through cognitive function was dependent on the moderator gender: much stronger in men than in women. Gender differences in cognitive function have been well documented [42]. Nicola Veronese and colleagues found that gait speed was a predictor of depression in men, while the five‐time sit‐stand tests were a predictor of depression in women [29]. Brain imaging studies have found that men and women activate significantly different neural networks during brain‐rotation tasks, such as increased activation in the parietal lobe in men and increased activation in the frontal lobe in women [43, 44]. A study revealed that depression was a strong predictor of membership in the rapid functional decline trajectory group among men, whereas women in the same group were more likely to be obese. These findings suggest that programs aimed at preventing or delaying functional decline in older adults should be gender‐sensitive [45]. Perhaps, we can argue that gender differences in cognition lead to differences in the relationship between physical performance and depressive symptoms. This finding may reflect the profound influence of gender roles in Chinese society: traditionally, males shoulder the role of primary breadwinner in the family, and declining physical function after retirement may thus impose a greater impact on their self‐identity. In contrast, Chinese females generally maintain more active social networks and family roles, which may to some extent buffer the psychological impact of reduced physical capacity.

To the best of our knowledge, this study for the first time investigated the predictive effect of poor physical performance on future depressive symptoms in Chinese elderly: the mediating role of cognitive function, based on a cohort design. This model may be explained the mechanism behind the link between depressive symptoms and physical performance. However, this study has several limitations. First, because of only one follow‐up, time‐varying exposures were not included in this study, and residual confounding should be a concern. Second, limitations similar to other studies, depressive symptoms were self‐reported, which may cause information bias. Third, the database we used was not included some confounding factors of depressive symptoms, such as isolation, anxiety, income, and social support, which may affect the observed relationship. Last, this study focused on analyzing the predictive value of physical performance for depressive symptoms over a 3‐year period, with subsequent analyses using a cross‐lagged panel model to investigate their causal relationship.

## 5. Conclusion

Our study identified low physical performance as a significant predictor of depressive symptoms in older Chinese adults. Additionally, we found that cognitive function was separately and negatively correlated with depressive symptoms. Cognitive function also mediated the association between physical performance and depressive symptoms, and there are differences between men and women. These findings may help guide clinicians to better diagnose and manage depression in the context of concomitant poor physical performance and cognitive impairment. It also indicated that cognitive intervention in the poor physical performance of the population is beneficial to improve their depressive symptoms.

## Disclosure

The manuscript is approved by all authors for publication

## Conflicts of Interest

The authors declare no conflicts of interest.

## Author Contributions

Na Zhang: conceptualization, methodology, and writing–review and editing. Lu Chen: supervision. Manlan He: methodology and software. Fang Wang: software and validation.

## Funding

This work was supported by the National Natural Science Foundation of China (82471345), Clinical Trials from the Affiliated Drum Tower Hospital, Medical School of Nanjing University (2024‐LCYJ‐MS‐17), and Nanjing Drum Tower Hospital National Natural Science Foundation of China Youth Cultivation Project (2024‐JCYJ‐QP‐17).

## Data Availability

The data that support the findings of this study are available from the corresponding author upon reasonable request.
